# From Colonization to High Production and *Plasmodium vivax* Infection of *Anopheles darlingi* and *Anopheles deaneorum*: a Platform for Malaria Research

**DOI:** 10.21769/BioProtoc.5302

**Published:** 2025-05-05

**Authors:** Maisa S. Araujo, Alice O. Andrade, Alessandra S. Bastos, Najara Akira C. Santos, José Daniel C. Pontual, Jéssica E. Araújo, Marina L. Rocha, Maria Eduarda R. Miguel, Ana Eliza M. Costa, Joseph M. Vinetz, Ricardo T. Gazzinelli, Jansen F. Medeiros

**Affiliations:** 1 Plataforma de Produção e Infecção de Vetores da Malária (PIVEM), Laboratório de Entomologia, Fiocruz Rondônia, Porto Velho, Rondonia, Brazil; 2 Programa de Pós-Graduação em Conservação e uso de Recursos Naturais – PPGReN, Fundação Universidade Federal de Rondônia, Porto Velho, Rondonia, Brazil; 3 Laboratório de Pesquisa Translacional e Clínica, Centro de Pesquisa em Medicina Tropical, Porto Velho, Rondônia, Brazil; 4 Instituto Nacional de Epidemiologia da Amazônia Ocidental - INCT-EpI-AmO, Porto Velho, RO, Brasil; 5 Programa de Pós-Graduação em Saúde Pública, Faculdade de Saúde Pública, Universidade de São Paulo, São Paulo, Brasil; 6 Section of Infectious Diseases, Department of Internal Medicine, Yale School of Medicine, New Haven, CT, USA; 7 Laboratório de Imunopatologia, Instituto René Rachou, Fundação Oswaldo Cruz, Belo Horizonte, Minas Gerais, Brazil; 8 Department of Medicine, University of Massachusetts Medical School, Worcester, MA, USA

**Keywords:** Colony establishment, *Anopheles*, Plasmodium vivax, Rearing, Membrane feeding assay

## Abstract

The mass rearing of anopheline mosquitoes under laboratory conditions is essential for advancing malaria research. It facilitates in-depth studies on mosquito biology, behavior, and genetics and their role in *Plasmodium* transmission. However, the colonization of Neotropical anophelines such as *Anopheles darlingi*—a primary malaria vector in the Amazon region—has proven particularly challenging due to its unique reproductive characteristics. Unlike other species that can initially be colonized using forced copulation methods and later adapt to natural mating, *An. darlingi* does not copulate under forced conditions. Recent breakthroughs in *An. darlingi* colonization have been achieved using flashlight induction techniques, which have enabled the establishment and maintenance of stable laboratory populations. These advancements have created new opportunities for vector control studies in Brazil, including the testing of innovative control methods and *Plasmodium* transmission-blocking strategies. This protocol offers a comprehensive, step-by-step guide for initiating and scaling up large laboratory colonies of *An. darlingi* and *An. deaneorum*, a secondary malaria vector. It details methods for copulation induction, colony management, and successful artificial infection of mosquitoes with *Plasmodium vivax*. The guide serves as a critical resource for establishing new Neotropical anopheline colonies from different populations, contributing to future malaria research and control efforts in the Amazon. Additionally, the establishment of Brazil’s first Malaria Vector Production and Infection Platform (*Plataforma de Produção e Infecção de Vetores da Malária*, PIVEM) has further supported the development of new control technologies and the study of *P. vivax–Anopheles* interaction, advancing efforts to combat malaria in the region.

Key features

• High production and experimental infection of *Anopheles* by *Plasmodium vivax.*

**This protocol is used in:** Rev Soc Bras Med Trop (2019), DOI: 10.1590/0037-8682-0159-2019; Mem Inst Oswaldo Cruz (2020), DOI: 10.1590/0074-02760200070; Front Microbiol (2022), DOI: 10.3389/fmicb.2022.971083; Sci Rep (2023), DOI: 10.1038/s41598-023-44556-y; Am J Trop Med Hyg (2024), DOI: 10.4269/ajtmh.23-0349

## Graphical overview


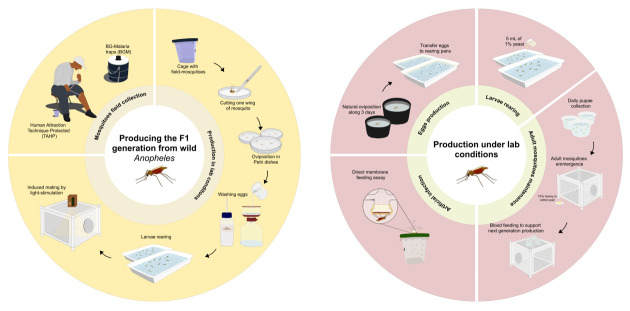


**Graphical overview of laboratory procedure for F1 generation and maintenance of *Anopheles darlingi* and *Anopheles deaneorum* colonies.** The procedure for producing the F1 generation begins with the capture of wild mosquitoes using the human attraction technique protected (TAHP) and BG-malaria traps (BGM). Captured females are induced to oviposit in Petri dishes following the first gonotrophic cycle under lab conditions. Egg hatching is stimulated by washing the eggs with a 0.25% hypochlorite solution. The larvae are reared to the pupae stage, after which the pupae are transferred to large cages until adults emerge. Once the laboratory colony achieves natural oviposition and mating, the maintenance procedures are streamlined to focus on egg production, larval rearing, and adult mating. Experimental infections with *Plasmodium vivax* parasites are performed using direct membrane feeding assays (DMFA).

## Background

The mass rearing of *Anopheles* mosquitoes in laboratories is essential for malaria research, enabling controlled studies on their biology, behavior, and vector competence. While *Anopheles gambiae* sensu stricto (s.s.) has been extensively studied since its colonization in 1953 [1], key aspects of *Anopheles darlingi* (Root, 1926), the primary malaria vector in the Amazon region [2,3], remain poorly understood due to the absence, until recently, of sustainable laboratory colonies.

In 1990, an *Anopheles deaneorum* (Rosa-Freitas 1989) colony was first established using the force mating technique [4]; no other colony was successfully maintained until 2020 [5]. This species serves as a secondary vector in certain Amazonian regions and has the potential to expand its geographic range due to climate change [6,7].

Historically, establishing *An. darlingi* colonies under laboratory conditions has proven challenging. Attempts to establish *An. darlingi* colonies date back to 1947, with colonization in the Cooperative Republic of Guyana (formerly British Guiana), Colombia, and Brazil [8–10]. Additional colonies were reported in Brazil in 1970 and 1988 [11,12], but long-term maintenance proved difficult. After nearly 40 years, new free-mating *An. darlingi* colonies were successfully established in Peru [13,14], Brazil [15], and in French Guiana [16].

The reproductive characteristics of some *Anopheles* species, such as eurigamy (the ability to mate freely in natural environments), initially limited their colonization in laboratories. However, this challenge was overcome by the development of the force mating technique, first described by [17] and later adapted by [18] and [19]. Larger cages or rooms were also used to facilitate mating swarms [11,12]. After a few generations, the natural selection of stenogamous strains allowed colonies of some *Anopheles* species to be maintained in smaller cages without the need for forced copulation. However, attempts to establish *An. darlingi* colonies using force mating techniques were largely unsuccessful.

Earlier attempts to colonize *An. darlingi* utilized larger cages or rooms and artificial lights to induce mating swarms [11,12]. Initial efforts resulted in colonies lasting only six generations [8], while later studies achieved maintenance for up to ten years [8,11], demonstrating the potential for long-term colonization despite challenges such as low sexual activity and oviposition rates [12]. Since 2010, *An. darlingi* colonies have been successfully established using induced copulation by light flashes, first described for *An. pseudopunctipennis* [20], with modifications such as reduced room temperature during inductions. These colonies, maintained for more than six generations, provide crucial insights into the species’ biology and support vector control strategies in the Amazon region. They also enable the testing of insecticides and population suppression or replacement technologies, such as the sterile insect technique (SIT), transgenic mosquito production, and release of insects carrying a dominant lethal gene (RIDL). Additionally, paratransgenesis techniques, which utilize symbiotic bacteria like *Asaia*, are being explored as potential malaria control strategies.

A recent study demonstrated that under laboratory conditions, the bacterium *Delftia tsuruhatensis*, found in the gut of *Anopheles* mosquitoes, can block the development of *P. falciparum* in *An. gambiae* s.s. through the production of the metabolite harmane [21]. Studies on the microbiota of *An. darlingi*, including its midgut and salivary glands, are currently underway to identify symbiotic bacteria suitable for paratransgenesis [22].

In Brazil, the establishment of *An. darlingi* [15] and *An. deaneorum* [5] colonies, coupled with successful *P. vivax* infections, led to the creation of the first Malaria Vectors Production and Infection Platform (*Plataforma de Produção e Infecção de Vetores da Malária*, PIVEM) in 2020. PIVEM supports research on *Anopheles–Plasmodium* interactions, including *P. vivax* liver studies [23,24] and transmission-blocking interventions [25–29].

Here, we provide a comprehensive description of the procedures required to initiate and maintain the large-scale mass rearing of Neotropical *Anopheles* mosquitoes using flashlight-induction copulation. This step-by-step guide outlines the processes for colony establishment, scaling up production, and maintaining laboratory populations, including successful artificial infection with *P. vivax*.

## Materials and reagents

**A. Producing the F1 generation from wild *Anopheles darlingi* and *Anopheles deaneorum***

1. Foam box 50 L

2. Foam box 10 L

3. Foam box 5 L

4. Damp towels

5. Black plastic garbage bag 50 L

6. Spherical plastic cups with lid (750 mL) covered by mesh netting and secured with rubber bands and a plastic lid (here called field cages)

7. Cotton pad

8. Sucrose, common table sugar (*Açúcar Cristal Barralcool*) with a minimum concentration of 99.7%

9. Chicken (live adult *Gallus gallus domesticus*, 2–3 kg weight)

10. Ice

11. Dichotomous keys proposed by Consoli & Lourenço-de-Oliveira [30]

12. Qualitative filter paper 80 g, 90 mm (Unifil, catalog number: 501.009)

13. Qualitative filter Paper 80 g, 50 cm × 50 cm (Unifil, catalog number: 501.1250)

14. Feather-tip forceps (Bioquip, catalog number: 4748)

15. Dissection forceps (Fine Science Tools Inc., Dumont, #5-Fine)

16. Petri dish (Gosselin^TM^, catalog number: BP93B-101, diameter: 90 mm, height: 14.2 mm)

17. Distilled water

18. 0.22 μm vacuum filtration system (Kasvi, catalog number: K15-1000)

19. 10% sodium hypochlorite (Cinord, 69, https://www.primecirurgica.com.br/hipoclorito-de-sodio-10-kit-com-4-galaos-de-5-litros-cinord-p2816/p)

20. White plastic pans (30.3 × 22.1 × 7.5 cm) (here called rearing pans)

21. Squirt bottle 500 mL

22. Spherical plastic containers (500 mL) (here called pupae containers)

23. Black plastic container (800 mL, 15 × 20 × 4 cm) (here called oviposition containers)

24. Laboratory test sieve (63, 125, and 250 μm) (Bertel, catalog numbers: 350500, 405983, and 500781)

25. Brush size 0

26. Beaker 5 mL, graduated glass

27. Tetra Marine Granules (Tetra^®^)

28. 3 mL plastic Pasteur pipette

29. Yeast extract power, 100 g (Chemical, catalog number: 8013-01-2 or similar)

30. Tube for mixing yeast, e.g., 50 mL disposable

31. Honey solution (local production, Apicultura Colonial, Vilhena, Rondonia)

32. 1 L clear plastic pitchers with volume markings

33. Sieves of different meshes (commercial)

34. Cages (30 × 30 cm) in aluminum (Horst Armadilhas Ltda-Me, http://www.horstarmadilhas.com.br/)

**B. Induced mating by light stimulation to natural copulation**

1. Cages (61 × 61 cm, 46 × 46 cm and 30 × 30 cm) in aluminum (Horst *Armadilhas Ltda-Me*, http://www.horstarmadilhas.com.br/)

2. Black plastic container (800 mL, 15 × 20 × 4 cm) (here called oviposition containers)

3. Chicken (live adult *Gallus gallus domesticus*, 2–3 kg weight)

4. Cotton pad

5. Honey solution (Local production, Apicultura Colonial, Vilhenal Rondonia)

6. Qualitative filter paper 80 g, 50 cm × 50 cm

**C. *Anopheles darlingi* and *Anopheles deaneorum* rearing and maintenance for scientific experiments**

1. Swine sodium heparin 5,000 U.I./mL (HEPAMAX-S, catalog number: 22021550)

2. Bovine blood

3. Glass bottle, 500 mL

4. Qualitative filter paper 80 g, 50 cm × 50 cm (Unifil, catalog number: 501.1250)

5. Distilled water

6. Squirt bottle 500 mL

7. White plastic pans (30.3 × 22.1 × 7.5 cm) (rearing pans)

8. White plastic containers spherical (500 mL) (pupae containers)

9. Black plastic container (800 mL, 15 × 20 × 4 cm) (oviposition containers)

10. Laboratory test sieve (63, 125, and 250 μm) (Bertel, catalog numbers: 350500, 405983, and 500781)

11. Brush size 0

12. Lab spatula spoon size ø 5mm

13. Beaker 5 mL, graduated glass

14. Tetra Marine Granules (Tetra^®^)

15. 3 mL plastic Pasteur pipette

16. Yeast extract power, 100 g (Chemical, catalog number: 8013-01-2 or similar)

17. Tube for mixing yeast, e.g., 50 mL disposable

18. Honey solution (local production, *Apicultura Colonial, Vilhena, Rondonia*)

19. 1 L clear plastic pitchers with volume markings

20. Sieves of different meshes (commercial)

21. Cages (30 × 30 cm) in aluminum (Horst *Armadilhas Ltda-Me*, http://www.horstarmadilhas.com.br/)

22. Styrofoam cups (180 mL size)

23. Polytetrafluoroethylene (PTFE) seal tape (~80 × 20 × 0.2 mm)

**D. Artificial infection by direct membrane feeding assay (DMFA)**

1. 6 mL vacuum sodium heparin tubes (BD Vacutainer, catalog number: 367886)

2. 1,000 μL to 200 μL pipette and tips

3. Human AB serum inactivate (human donation)

4. Parafilm “M” roll, 10.16 cm × 38.10 (American, catalog number: PM996)

5. Spherical plastic cups (650 mL) covered by mesh netting and secured with rubber bands and a plastic lid (blood feeding cups)

6. Spherical plastic cups (2,000 mL) covered by mesh netting and secured with rubber bands and a plastic lid (infected cups)

7. 70% ethanol in water

8. Honey solution (local production, *Apicultura Colonial, Vilhena, Rondonia*)

9. Cotton pad

**E. Midgut and salivary gland dissection**

1. Glass slides 26 × 76 mm, 1–1.2 mm thickness (Perfecta^TM^, catalog number: 7105)

2. Coverslips 13 mm, 0.13–0.16 mm thickness (Olen^TM^, catalog number: K5-0013)

3. Dissection needle 0.20 mm (Rodoz Surgical Instrument CO., catalog number: RS-6083-20)

4. Needle holder for micro dissecting needles (Rodoz Surgical Instrument CO., catalog number: RS-6061)

5. Dissection forceps (Fine Science Tools Inc., Dumont, #5-Fine)

6. Mercury dibromofluorescein disodium salt (Mercurochrome, Sigma-Aldrich, catalog number: M7011-10G)

7. RNA-free disposable pellet pestles (Fisherbrad^TM^, catalog number: 12-544-2)

8. DNA LoBind tube 1.5 mL (Eppendorf^ TM^, catalog number: J190474)

9. PBS solution

10. Glass or plastic pestle for 1.5 mL tubes (Merck^TM^, catalog number: BAF199230001)

11. Neubauer chamber (Olen, catalog number: K5-0111)

12. RPMI-1640 medium (Sigma-Aldrich, catalog number: R5886-100ML)

**F. Cleanliness and general maintenance**

1. 10% Sodium hypochlorite (Cinord, 69, https://www.primecirurgica.com.br/hipoclorito-de-sodio-10-kit-com-4-galaos-de-5-litros-cinord-p2816/p)

2. 100% lab detergent (Extran MA02 Neutro Merck 5 L or similar)

3. 15% peracetic acid (Spartan^®^, https://www.spartanbrasil.com.br/produtos/detalhes/83/peraceticfood.html#info)

4. Ethanol (absolute)

**Solutions**

1. 10% sucrose solution (see Recipes)

2. 0.25% sodium hypochlorite (bleach) solution (see Recipes)

3. 0.5% sodium hypochlorite (bleach) solution (see Recipes)

4. 15% honey solution (see Recipes)

5. 1% yeast solution (see Recipes)

6. 70% ethanol (see Recipes)

7. 2% mercurochrome stock solution (see Recipes)

8. 0.2% mercurochrome solution (see Recipes)

9. 6% lab detergent (see Recipes)

10. 0.02% peracetic acid solution (see Recipes)

**Recipes**

**1. 10% sucrose solution**

**Table:** 

Reagent	Final concentration	Amount
Sucrose	10%	5 g
H_2_O	n/a	Fill to 50 mL
Total	n/a	50 mL

Keep up to 7 days stored at 4 °C. Leave for 1 h at room temperature before use.

**2. 0.25% sodium hypochlorite (bleach) solution**

**Table:** 

Reagent	Final concentration	Amount
Sodium hypochlorite solution (10%)	0.25%	75 mL
ddH_2_O	n/a	Fill to 3,000 mL
Total	n/a	3,000 mL

Keep up to 7 days; store at room temperature.

**3. 0.5% sodium hypochlorite (bleach) solution**

**Table:** 

Reagent	Final concentration	Amount
Sodium hypochlorite solution (10%)	0.5%	50 mL
ddH_2_O	n/a	Fill to 1,000 mL
Total	n/a	1,000 mL

Keep up to 7 days; store at room temperature.

**4. 15% honey solution**

**Table:** 

Reagent	Final concentration	Amount
Honey	15%	150 mL
ddH_2_O	n/a	Fill to 1,000 mL
Total	n/a	1,000 mL

Keep up to 7 days stored at 4 °C. Leave for 1 h at room temperature before use.

**5. 1% yeast solution**

**Table:** 

Reagent	Final concentration	Amount
Yeast	1%	10 g
ddH_2_O	n/a	Fill to 1,000 mL
Total	n/a	1,000 mL

Keep up to 7 days stored at 4 °C. Leave for 1 h at room temperature before use.

**6. 70% ethanol**

**Table:** 

Reagent	Final concentration	Amount
Ethanol (absolute)	70%	700 mL
H_2_O	n/a	Fill to 1,000 mL
Total	n/a	1,000 mL

Store at room temperature.

**7. 2% mercurochrome stock solution**

**Table:** 

Reagent	Final concentration	Amount
Mercurochrome	2%	0.2 g
H_2_O	n/a	Fill to 10 mL
Total	n/a	10 mL

Store at room temperature.

**8. 0.2% mercurochrome solution**

**Table:** 

Reagent	Final concentration	Amount
Mercurochrome 2%	0.2%	1 mL
H_2_O	n/a	Fill to 10 mL
Total	n/a	10 mL

Store at room temperature.

**9. 6% lab detergent**

**Table:** 

Reagent	Final concentration	Amount
Lab detergent 100%	6%	60 mL
H_2_O	n/a	Fill to 1,000 mL
Total	n/a	1,000 mL

Store at room temperature.

**10. 0.02% peracetic acid solution**

**Table:** 

Reagent	Final concentration	Amount
Peracetic acid solution 15%	0.02%	700 μL
H_2_O	n/a	Fill to 500 mL
Total	n/a	500 mL

Store at room temperature.

## Equipment

**A. Producing the F1 generation from wild *Anopheles darlingi* and *Anopheles deaneorum***

1. BG-malaria traps (BGM) [31]

2. Mouth aspirator (Castro type) (Horst *Armadilhas Ltda-Me*, http://www.horstarmadilhas.com.br/)

3. InsectaVac vacuum-powered insect aspirator (BioQuip Products, catalog number: 2809B)

4. Stereomicroscope (Leica, model: EZ4)

5. Suction filtration vacuum pump (Nevoni, model: MOD5005)

**B. Induced mating by light stimulation to natural copulation**

1. Blue stroboscopic light source (Opaluz strobe warming light 30 W)

2. InsectaVac vacuum-powered insect aspirator (BioQuip Products, catalog number: 2809B)

**C. *Anopheles darlingi* and *Anopheles deaneorum* rearing and maintenance for scientific experiments**

1. Hemotek membrane feeding system used with Parafilm membrane (Hemotek^®^, catalog number: SP6W1-3)

2. Newbauer chamber (Olen, catalog number: K5-0111)

3. Centrifuge 5702 (Eppendorf^®^, catalog number: 5703000322)

4. InsectaVac vacuum-powered insect aspirator (BioQuip Products, catalog number: 2809B)

5. Stereomicroscope (Leica, catalog number: EZ4)

6. Microscope (Leica, catalog number: DM750)

## Procedure

**A. Producing the F1 generation from wild *Anopheles darlingi* and *Anopheles deaneorum***

1. Obtaining field-caught *An. darlingi* and *An. deaneorum*

a. Collect approximately 200–300 adult females using BG-malaria traps and/or human attraction technique protected (TAHP) in a peridomicile malaria-endemic area from 6 p.m. to 9 p.m. [5,15].

b. Transfer all collected mosquitoes into plastic cups covered with mesh netting (field cages), with 30–50 mosquitoes per cage. Maintain them with moistened cotton balls soaked in 10% sucrose solution, covered with damp towels and a black plastic garbage bag. Store the cages in a 50 L foam box for transport to the lab.

2. Production of mosquitoes in lab conditions

a. On the day following mosquito collection, remove the sucrose solution 6 h before blood feeding to induce fasting. For blood feeding, physically restrain the chicken on top of the cage, allowing the mosquitoes to feed for 15 min.

*Note: The best time for blood feeding is at the end of the day, around 5–6 p.m.* (GTM -4:00).

**Figure 1.**
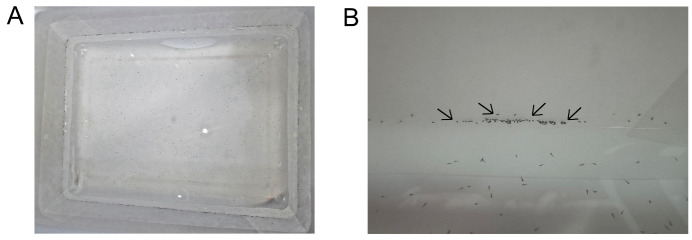
Eggs transferred to rearing pans. (A) Rearing pans lined with filter papers and filled with 1 L of distilled water containing eggs. (B) *Anopheles darlingi* eggs adhered to the filter paper lining the rearing pan walls. The black arrows show the eggs adhered to the wall of the rearing pans.

b. On the fourth day after blood feeding, anesthetize the mosquitoes by placing the cups containing them in a 5 L foam box with ice for 15 min. Identify the mosquito species under a stereomicroscope, using the dichotomous identification keys described by Consoli and Lourenço-de-Oliveira [30].

c. Mosquito species considered important malaria vectors, such as *An. darlingi* and *An. deaneorum*, should undergo induced oviposition. To do this, use dissection tweezers to remove one wing and place the mosquito without one wing in a Petri dish lined with moistened filter paper (80 g, 90 mm). Place five mosquitoes per Petri dish and store them in a 10 L foam box, maintaining insectary conditions.

d. Daily, transfer live mosquitoes to new Petri dishes lined with moistened filter paper, discard the dead mosquitoes, and moisten the Petri dishes containing eggs using a 3 mL plastic Pasteur pipette. Repeat this procedure for three days before proceeding with egg washing.

e. To wash the eggs, use a 0.22 μm vacuum filtration system that is connected to a vacuum pump. Transfer all eggs from the filter paper to the vacuum filtration system using a 500 mL squirt bottle filled with distilled water. Perform an initial filtration to remove the excess water and then the eggs by adding 1 L of 0.25% hypochlorite and filtering after 30 s. Repeat this process three times, alternating with the addition of 1 L of distilled water.

f. After washing the eggs, transfer them to rearing pans with the walls lined with filter paper and filled with 1 L of distilled water. The quantity of eggs should be sufficient to cover all the walls of the rearing pans (see Figure 1).

*Note: At the end of each day, use a wash bottle to squirt water onto the walls of the rearing pans to stimulate hatching*.

g. On the day following egg washing, begin larval feeding using TetraMarine Granules^®^, putting the food with a brush. See Table 1 for feeding details.

**Table 1.** Larvae feeding schedule and preparation

Instar	Food preparation	Feeding frequency	Use
1	Ground and sieved through 63 μm	Once per day	Brush
2	Ground and sieved through 125 μm	Twice per day	Brush
3	Ground and sieved through 250 μm	Three times per day	Spatula spoon
4	Ground and sieved through 250 μm	Three times per day	Spatula spoon

h. On the third day after transferring eggs to rearing pans, add 5 mL of 1% yeast solution at the end of the day using a 5 mL beaker. On the fifth day, change the water in the larval rearing pans, transferring the larvae to a clean pan with 200 larvae per pan.

*Note: After this first water change, subsequent water changes should be performed every Monday, Wednesday, and Friday*.

**Critical step:** During water changes, larvae should be separated by instar into separate pans using sieves of different mesh sizes (see Video 1).

**Video 1.**
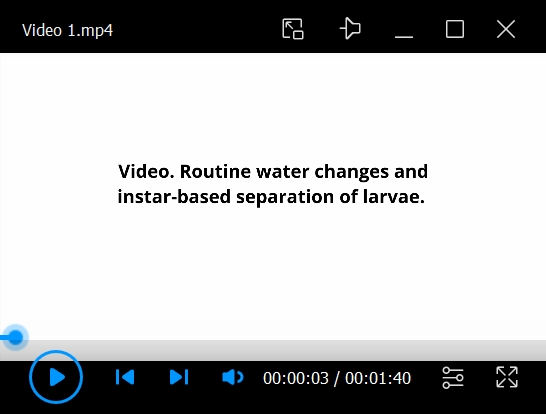
Routine water changes and instar-based larvae separation

i. Around 10 days after egg washing, pupae should be removed daily using a plastic Pasteur pipette (3 mL plastic) and transferred to pupal containers inside a screened cage (30 cm × 30 cm) for adult emergence.

j. Adult mosquitoes should be maintained on a 15% honey solution under controlled conditions: 26 ± 1 °C, 70% ± 10% relative humidity, and a 12/12 h day/night photoperiod.

**B. Induced mating of *Anopheles darlingi* and *Anopheles deaneorum* using light stimulation**

1. When 1,500–2,000 mosquitoes aged 3–5 days are available, transfer approximately 800–2,000 male and female mosquitoes (ratio 1:1) into screened cages (61 cm × 61 cm × 61 cm) using a Castro aspirator or vacuum-powered insect aspirator. Maintain mosquitoes on 15% honey solution ad libitum.

2. Six hours before copulation induction, remove the honey solution and adjust insectary conditions by reducing the temperature to 24 ± 1 °C and setting a night photoperiod.

3. Begin copulation induction at 6 p.m. by exposing the cage to a light beam (white 5 mm LEDs, 6,000K, 18,00 0MCD, 2 Hz pulse) for four cycles of 10 min on and 10 min off. Position the light source directly above the cage, 61 cm from the bottom, to ensure top-down illumination. Repeat copulation induction for 5–7 consecutive evenings.

*Notes:*

*1. Light cycles can be performed manually using a flashlight [15] or with an automated system as described by Araujo et al. [5].*

*2. Copulation will begin immediately upon flashlight activation, and mosquito mating pairs will be observed falling to the bottom of the cage (see Video 2).*

**Video 2.**
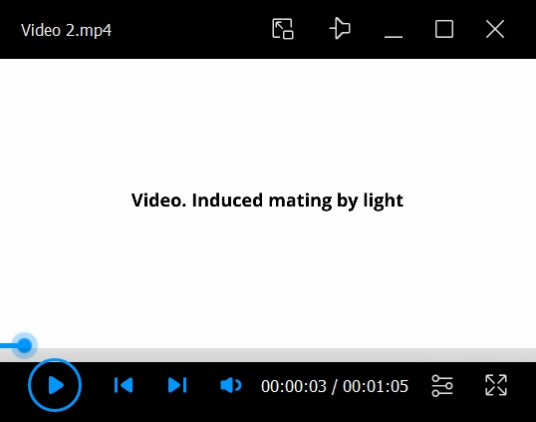
Induced mating by light

4. Following the final light cycle, feed female mosquitoes on chicken blood for 15 min during the first 4–6 days of induction. Restore normal insectary conditions (temperature, relative humidity, and photoperiod) as described in step A2j.

5. Up to the F5–F6 generation, copulation induction should be performed for each generation. After that, natural mating will occur at the transition from light to dark (6 p.m.).

6. For natural copulation, maintain 3–5-day-old mosquitoes under lab conditions for three days. On the fourth day, feed mosquitoes on chicken blood for 15 min to initiate the gonotrophic cycle.

7. Induction oviposition should be performed as described in step A2b–i. This procedure is required until the F5–F6 generation, after which natural oviposition will occur when oviposition containers are placed inside the cages.

8. For natural oviposition, place four oviposition containers (walls covered with filter paper and about 200 mL distilled water) inside the cages 48 h after blood feeding.

9. From the F8 generation, mosquitoes should be adapted to smaller cages. Begin with 46 cm × 46 cm cages, then transition to 30 cm × 30 cm cages for colony maintenance.

**C. *Routine* maintenance of *Anopheles darlingi* and *Anopheles deaneorum* laboratory colony**

See the suggested schedule for routine maintenance in Table S1.

1. Eggs collection and handling

a. Remove oviposition containers from cages three days after blood feeding and transfer eggs to rearing pans.

*Notes:*

*1. Eggs should be produced weekly from blood-fed mosquitoes.*

*2. Two oviposition containers per cage are sufficient to maintain colony production.*

b. Before transferring eggs, remove all dead mosquitoes from the oviposition container. Prepare a transition pan by covering it with filter paper and transferring all water from the oviposition container into this pan.

c. Wait for 1 min to allow eggs to adhere to the filter paper. Use one filter paper to seed two rearing pans for first-instar larvae.

*Notes:*

*1. Rearing pans for first-instar larvae should have walls covered with filter paper*.

*2. Identify all pans with information about the generation and transfer date of eggs.*

*3. Typically, one oviposition container produces six rearing pans for first-instar larvae*.

*4. Once all first-instar rearing pans are prepared and placed on shelves, squirt water onto the pan walls using a wash bottle to stimulate hatching*

*5. Clean and discard water from oviposition containers following Section F (cleanliness and general maintenance).*

d. Larvae feeding will begin only on day one after egg transfer, following Table 1.

e. Subsequent steps of the procedures are the same as described in step A2h.

2. Larvae rearing

a. Initiate larval feeding on day one post-hatching (Table 1). Rear ~600 first-instar larvae per pan, using 1 L of distilled water.

b. On day two, add 5 mL of 1% yeast solution at the end of the day to improve larval feeding.

c. On day four (Saturday; see suggested schedule for routine maintenance in Table S1), transfer larvae to new pans with fresh water, without filter paper. Larvae density should be 300 larvae per pan until the next water change.

*Note: Dirty pans must be cleaned, and discarded water must follow the procedures described in Section F (cleanliness and general maintenance).*

d. After that, the water change routine follows this schedule:

Monday: Change water in first and second-instar larvae rearing pans.

Friday: Change water in all rearing pans, except newly transferred rearing egg pans.

*Notes:*

*1. On water change days, add 5 mL of 1% yeast solution at the end of the day*.

*2. Identify rearing pans with the following information: generation, egg transfer date, and water change date.*

*3. Water changes are crucial for separating the larvae by instar using sieves of different mesh sizes or a Pasteur pipette if necessary.*

*4. Maintain a density of 250 larvae per pan for second- to fourth-instar larvae.*

*5. Larval development typically takes 9–15 days, after which pupae appear.*

*6. Maintain the water temperature of rearing pans at 24 ± 1 °C (see Table S2 for temperature registers).*

3. Pupae management

a. Collect pupae daily using a Pasteur pipette and transfer them to a pupae container (holding 300–500 pupae) inside a rearing cage (30 cm × 30 cm) with a cotton pad soaked in 15% honey solution.

*Notes:*

*1. A form and identification badge related to the cage and registration of pupae production and mortality should be recorded (Tables S3 and S4).*

*2. Do not add pupae older than 5 days to a single cage to avoid sex ratio imbalances (males typically pupate first).*

4. Adult mosquito maintenance

a. Maintain adult mosquitoes on 15% honey solution ad libitum. Replace the cotton pad with fresh honey solution every Tuesday, Thursday, and Saturday (see suggested schedule for routine maintenance in Table S1).

b. Recorded dead pupae (use the form in Table S3) on the cotton pad replacement days. Once all pupae have emerged, remove pupae containers from cages.

c. Separate cages containing 3–5-day-old mosquitoes for blood feeding to produce the next generation.

d. On Wednesday (see suggested schedule for routine maintenance in Table S1), remove the honey solution at 8 a.m. and initiate blood feeding at 5:30 p.m. (GTM -4:00).

e. For colony maintenance, blood feeding is performed using bovine blood, obtained fortnightly from a local slaughterhouse, collected in 30 U.I./mL swine sodium heparin [32].

*Note: On bovine blood collection day, prepare a 500 mL glass bottle containing 30 U.I/mL swine sodium heparin (600 μL of anticoagulant to every 100 mL of bovine blood). Store blood at 4 °C for up to 7 days.*

f. Prepare the artificial feeding system by inverting the Styrofoam cups and adding 3 mL of bovine blood to the bottom surface of each cup (four cups per cage). Subsequently, cut a small piece of PTFE membrane and stretch it over the blood droplet to facilitate mosquito feeding.

g. Maintain blood temperature at ~37 °C by adding warm water to the cups. Replenish warm water as needed to maintain temperature at 37 °C. Allow mosquitoes to feed for 30 min.

h. After blood feeding, return the cotton pad soaked in 15% honey solution to the cage. Discard used Styrofoam cups following Section F (cleanliness and general maintenance).

i. Record the blood-feeding date on the cage identification badge and blood feeding form (Supplementary S4 and S5).

j. Seventy-two hours post-blood feeding (Saturday; see suggested schedule for routine maintenance in Table S1), introduce 2–4 oviposition containers (walls lined with filter paper, each containing 200 mL of distilled water) into each cage.

k. Record the oviposition container introduction date on the cage identification badge and oviposition form (Tables S4 and S5).

**D. *Plasmodium* infection of *Anopheles darlingi* and *Anopheles* mosquitoes by direct membrane feeding assay (DMFA)**

Mosquito infections requiring a high number of oocysts or high prevalence rates may be enhanced by replacing plasma with heat-inactivated naïve human AB^+^ serum, as detailed in steps D2 and D3. If these criteria are unnecessary, proceed experimental infection directly from step D1 to step D4.

*Note: All infection procedures must adhere to strict biological safety regulations. This includes proper use of personal protective equipment (PPE), employment of appropriate containment devices, and compliance with institutional laboratory safety protocols. All infection procedures described herein follow establish institutional laboratory safety practices.*

**Figure 2.**
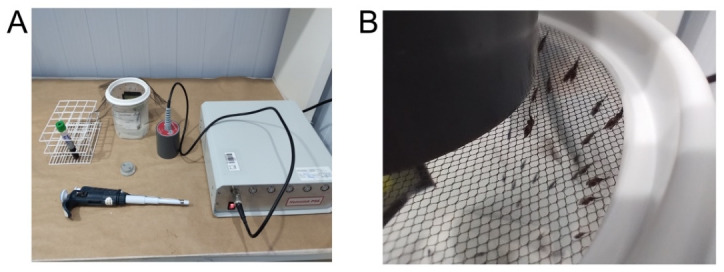
*Plasmodium* infection of mosquitoes by direct membrane feeding assays (DMFA). (A) Blood feeding using the Hemotek^®^ system. (B) Female *Anopheles darlingi* mosquitoes feeding on infected blood.

1. After obtaining informed consent, collect *P. vivax*–infected blood from symptomatic patients via venipuncture into heparinized tubes. Maintain blood samples at 37 °C throughout all manipulations using a water bath or thermos bottle.

2. Centrifuge blood at 500× *g* for 5 min at 37 °C.

3. Carefully remove patient plasma and white blood cells, replacing them with heat-inactivated human AB^+^ serum from healthy donors at a 1:1 ratio (56 °C for 30 min) [24,33].

4. Pipette 1,000 μL of the prepared blood-serum mixture, or whole blood sample, into the blood reservoir lined with parafilm membrane (20 cm × 20 cm piece) and connect it to the feeding station (Figure 2).

5. Prepare 60–100 female mosquitoes (aged 3–5 days, fasted for at least 12 h) for blood feeding inside a feeding cup.

6. Allow female mosquitoes to engorge for 30 min.

7. Post-feeding, anesthetize mosquitoes by placing the feeding cups in a foam box with ice for 1–2 min. Remove the mesh netting and visually separate fed females (enlarged, darkened abdomen) from unfed mosquitoes.

8. Euthanize all non-engorged mosquitoes by transferring them into a 100 mL glass beaker containing 70% ethanol.

9. Transfer engorged female mosquitoes to an infection cage, reattach the mesh netting, and maintain them under standard insectary conditions, as previously described, with 15% honey solution until dissection.

10. Day 7 post-infection, dissect the midguts (Video 3, see Section E) from 25–35 female mosquitoes to assess oocysts load. Day 14 post-infection, dissect salivary glands (Video 4, see Section E) from all remaining mosquitoes to assess sporozoite load.

**Video 3.**
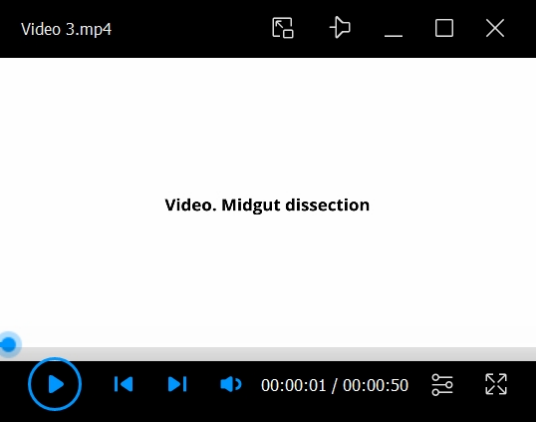
Dissection of midguts

**Video 4.**
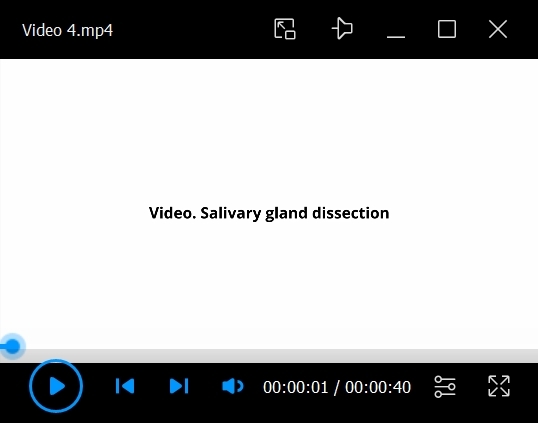
Salivary gland dissection

**E. Midgut and salivary gland dissection**

1. Anesthetize mosquitoes by placing the cages inside a foam box with ice for 1–2 min. Then, transfer anesthetized mosquitoes to a 100 mL glass beaker containing 70% ethanol by inverting the cage without mesh netting and quickly, but carefully, transferring the mosquitoes to a Petri dish containing 1× PBS using a forceps, grabbing them by the legs or wings to avoid damaging the head region.

*Notes:*

*1. Safety note: Ensure mosquitoes are fully anesthetized before transferring to a beaker with ethanol. Confirm that all mosquitoes are fully immersed in ethanol.*

*2. Safety note: Ethanol exposure kills mosquitoes but always use Petri dish lids to prevent accidental escape.*

**Figure 3.**
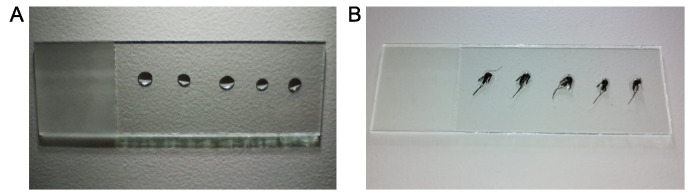
Mosquito dissection slide preparation. (A) Clean glass slide with five drops of 5 μL of 1× PBS. (B) Mosquitoes for dissection on the 1× PBS drops.

2. On the dissection bench (Figure 3), dispense 5–10 drops of 5 μL of 1× PBS onto a clean glass slide (one drop per mosquito). Prepare only one glass slide at a time.

3. Using fine forceps, grasp individual mosquitoes by the proboscis, leg, or wing and transfer each to a dissection buffer drop on the slide.

*Note: Mosquito positioning should accommodate the dissector’s handedness (right or left-handed).*

**Figure 4.**
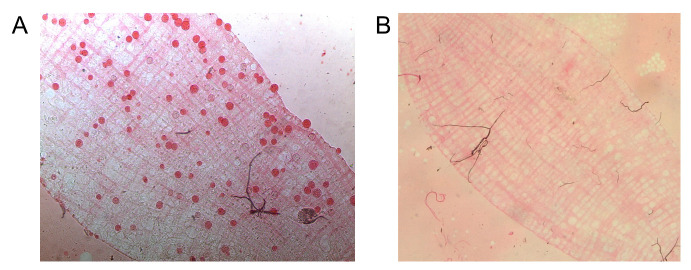
*Plasmodium vivax* oocyst in *Anopheles darlingi* midgut. (A) Infected midgut with 0.2% mercurochrome-stained oocysts (red). (B) Uninfected mosquito midgut control.

4. First, dissect the midgut using two 0.20 mm dissection needles, each mounted on micro dissecting needle holders. Stabilize the thorax with one dissection needle while simultaneously using dissection forceps to grasp the fifth abdominal segment and gently remove the mosquito abdomen in one continuous motion. Remove the thorax using the second dissection needle, then reposition this needle at the abdominal end to extract the complete midgut from the abdomen. Use both dissection needles to remove the Malpighian tubes (see Video 3).

5. Transfer midgut to a new glass slide with 3 μL of 0.2% mercurochrome, cover with coverslip, and wait 2 min before counting the number of oocysts at 10–20× magnification.

6. Count all oocysts (Figure 4) and record the number of oocysts per midgut.

7. To dissect salivary glands, follow steps E1 to E3.

8. With the proboscis oriented right, use two 0.20 mm dissection needles, each connected to a micro dissecting needle holder. Secure the head with one needle and the thorax with another. Gently pull the head rightward while stabilizing the thorax. The glands will remain connected to the head. Then, using the needle that secured the thorax, sever glands from the head and transfer them to collection tube.

**Figure 5.**
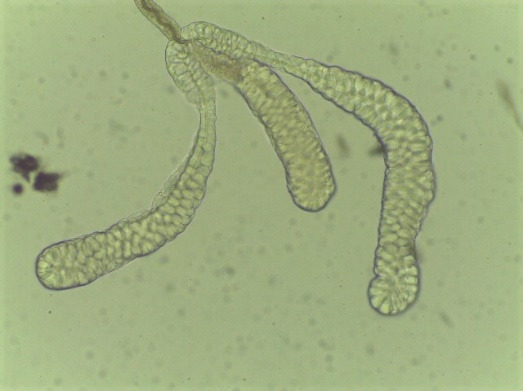
*Anopheles darlingi* salivary gland morphology. Paired organs, each gland containing three distinct lobes: two lateral and one medial lobe.

*Note: Intact salivary glands comprise three lobes (two lateral and one medial lobe; Figure 5).*

**Figure 6.**
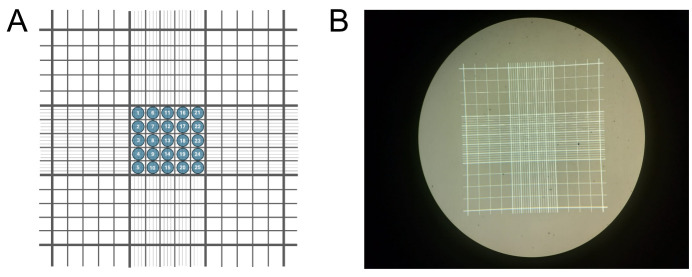
Neubauer chamber utilization. (A) Schematic representation of a Neubauer chamber, used for sporozoite counting. Counting is performed within the central 25 squares of the chamber. (B) Actual Neubauer chamber image.

9. Collect the glands by hooking them with needles and transfer them to a 1.5 mL DNA LoBind tube containing 15 μL of ice-cold RPMI-1640 medium. As salivary glands may adhere to the needle, ensure complete transfer under a stereomicroscope.

10. Process pools of 2–10 pairs of salivary glands per tube and then centrifuge the collection tubes for 20 s.

11. Use the matching pestle to crush the glands by rotating the pestle against the bottom of the 1.5 mL tube 30 times.

12. Re-centrifuge collection tubes for 20 s, then load 10 μL onto the Neubauer chamber and wait 10 min before counting sporozoites under a microscope.

13. Count sporozoites in 25 squares of the Neubauer chamber (Figure 6) and calculate the number of sporozoites per mosquito.

Count: (Count sporozoites/25) × 10,000/number of mosquitos in pool

**F. Cleanliness and general maintenance**

Maintaining rigorous cleaning conditions is essential for successful *Anopheles* colony production. These procedures prevent fungal and bacterial contamination while deterring pests such as ants. Typically, all rooms undergo daily cleaning, which involves mopping floors with a 0.5% hypochlorite solution to remove solid debris at the end of each workday. Additionally, a more thorough cleaning (general cleaning) is conducted once a month, including wall cleaning and washing the floor with 6% lab detergent. These tasks are performed by the institute’s cleaning team.

Furthermore, on the day of the general cleaning, platform technicians restock supplies, wash distillate water containers, sanitize equipment according to the manufacturer's instructions, and disinfect benches with 6% lab detergent.

1. Cleaning rearing pans, pupae, and oviposition containers

Clean materials using a soft sponge with 6% lab detergent and rinse with running water. After washing the materials, chemically sterilize them with 0.02% peracetic acid solution, applying it directly with a spray bottle to the inside of rearing pans and pupae and oviposition containers, allowing them to air dry at room temperature.

2. Distilled water container

*Anopheles* colonies from PIVEM require distilled water for their larval stages. The distilled water container is maintained inside the insectary at a controlled temperature and should be refilled daily.

The cleaning procedure follows the same steps as those for rearing pans, pupae containers, and oviposition containers. The distilled water container is washed weekly.

3. Dispose of rearing pan water after pupae collection or water change

Before discarding water, double-check to ensure no larvae are present. If larvae are found, transfer them to another rearing pan according to their generation and species. The water can then be discarded in the sink. For L1 and L2 instar larvae, which are particularly difficult to see, add boiling water to the pans, wait 2 min, and then discard the water down the sink.

4. Cage preparation

After using the mosquitoes, euthanize them and surface-sterilize the cage by spraying it with 70% ethanol. Remove the paper from the bottom containing the dead mosquitoes and discard it as biological waste. Clean the walls and bottom of mosquito cages with 70% ethanol and a paper towel. Once cleaned, line the bottom with a fresh paper towel cut to fit the cage dimensions.

5. Disposal of blood-feeding materials

All materials contaminated with human blood, serum, or infectious agents must be decontaminated before disposal following BSL-2 level guidelines.

Materials containing less than 2 mL of blood (e.g., glass feeders, Hemotek discs, tubes) should be decontaminated in a 1% bleach solution for at least 30 min. After decontamination, the solution can be disposed of in the sink, and solid waste (e.g., tubes, parafilm membrane) should be discarded in the biological waste bin. Feeders should be washed with neutral detergent using their designated brush under running water. Once dry, they should be stored in their designated locations.

Sharps waste (e.g., pipette tips, blades) must be disposed of immediately in a sharps container. Blood tubes containing more than 2 mL of blood should be placed in a designated, covered container for blood tubes, as they must be autoclaved before disposal. Blood-contaminated equipment (e.g., pipettes) should be cleaned with 1% bleach solution. Hemotek feeders should be cleaned with 70% alcohol followed by 1% bleach solution.

## Data analysis

The DMFA is performed using a single blood sample, which inherently limits the use of biological replicates. Consequently, each DMFA is treated as an independent experiment for data analysis. The key parameters evaluated in the DMFA include oocyst and sporozoite intensity, determined as the number of oocysts per midgut and the number of sporozoites per mosquito, respectively. Another parameter is the prevalence of infected mosquitoes, calculated as the percentage of infected mosquitoes, using the formula:

$$\%\;Prevalence\;rate=100\;\times \;\left(\frac{number\;of\;positive\;mosquitoes}{total\;of\;dissected\;mosquitoes}\right)$$

A positive mosquito is defined as one with at least one oocyst in its midgut. The total number of dissected mosquitoes refers to those examined at 7 days post-infection (dpi).

For data analysis, a preliminary statistical summary is performed for each DMFA. Zero values are excluded from the intensity of infection data. All raw oocyst and sporozoite count data are available in Tables S6 and S7, respectively. A summarized example of DMFA data can be found in Table S8. Following quality control, data that meet the inclusion criteria for infection prevalence and mean oocyst counts are used for further statistical analyses.

To assess the efficacy of transmission-blocking interventions, such as transmission-blocking vaccines (TBVs) and transmission-blocking drugs (TBDs) for *P. vivax*, Miura et al. [34] recommend using a transmission-reducing assay (TRA), which measures the percentage of reduction in oocyst density. This is preferred over a transmission-blocking assay (TBA), which evaluates inhibition in the prevalence of infected mosquitoes. The TRA readout is considered a better predictor of transmission-blocking intervention efficacy under field conditions, as a single oocyst can generate a significant number of sporozoites [35]. Miura et al. [33] recommended including only data where the mean oocyst count per midgut exceeds four in the TRA analysis. However, our analysis adopts a threshold of more than 2.5 oocysts per midgut [36]. For TBA evaluations, a prevalence rate of at least 60% is required for inclusion in statistical analyses.

**Figure 7.**
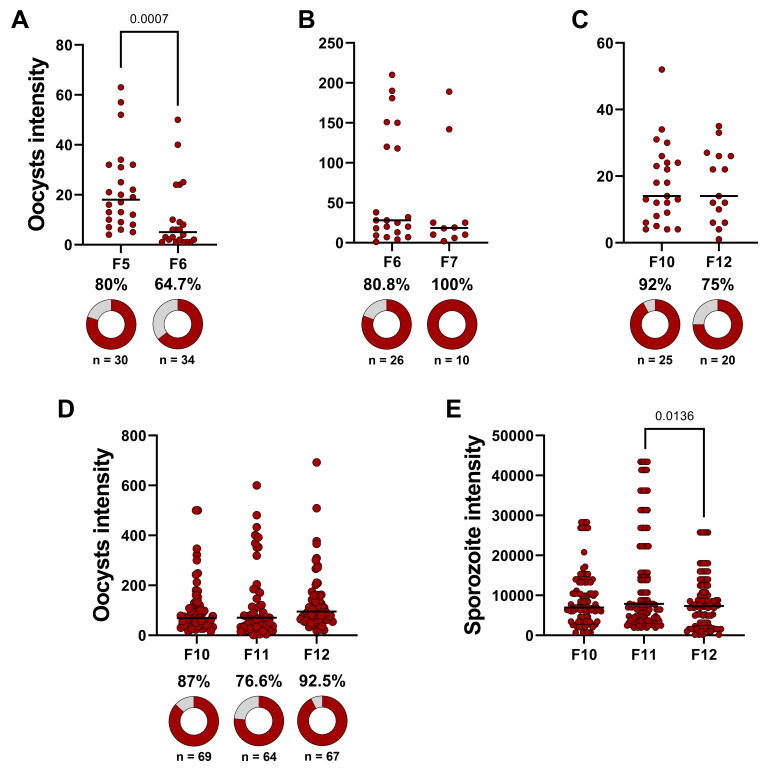
Data analysis evaluating *Anopheles darlingi*’s susceptibility to *Plasmodium vivax*. (A, B, and C) Oocyst intensity across different mosquito generations (F5 vs. F6, F6 vs. F7, and F10 vs. F12). A single direct membrane feeding assay (DMFA) was performed for each comparison, and statistical significance was assessed using the Mann–Whitney test. Donuts charts represent infection prevalence, with dark red segments indicating infected mosquitoes and gray segments indicating uninfected ones. The χ^2 ^test was used for statistical analysis. (D) Oocyst intensity and infection prevalence. (E) Sporozoite intensity. For panels D and E, four independent DMFA were performed, but DMFA 2757 was excluded from the analysis due to quality control protocols. Statistical comparisons among experimental groups were performed using the Kruskal–Wallis test with Dunn’s multiple comparisons. In all dot plots, each dark red circle represents a dissected mosquito, and horizontal bars indicate median values.

In Table S8 data, the DMFA identified as 2757 was excluded from statistical analysis due to a very low infection prevalence and mean oocyst count. After quality control, infected and uninfected mosquitoes within each DMFA are grouped, and infection prevalence is analyzed using a χ^2^ test. Bonferroni corrections are applied for comparisons involving more than two experimental groups. To evaluate oocyst intensity, grouped data from each DMFA are analyzed using the Mann–Whitney test for comparisons between two experimental groups or Kruskal–Wallis test with Dunn’s multiple comparisons for comparisons involving more than two groups. The same statistical approach is used for comparing sporozoite intensity. The results of the infection data analysis, detailed in Table S9, are presented in Figure 7.

## Validation of protocol

**Figure 8.**
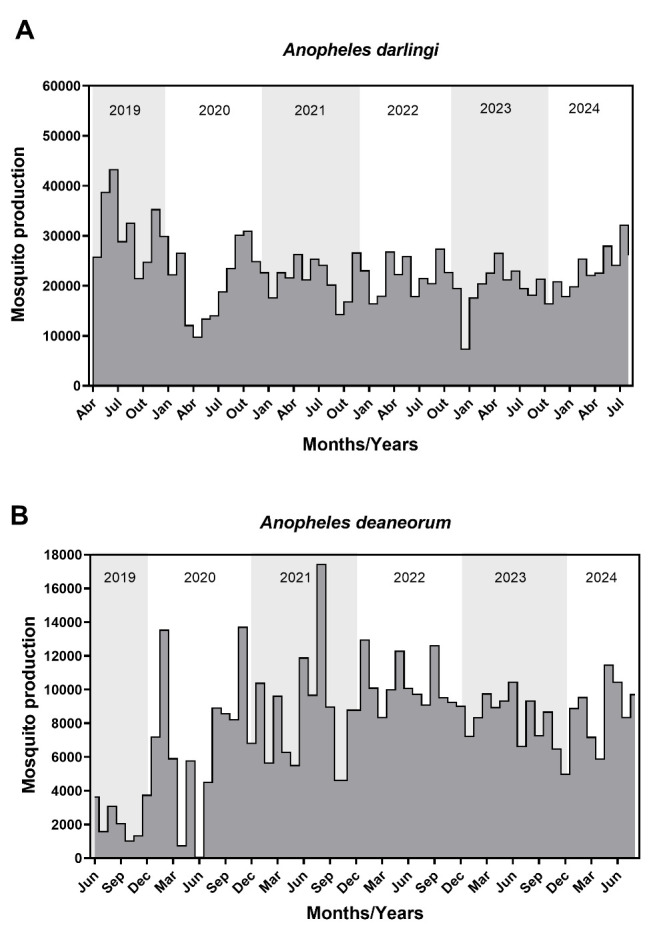
Adult mosquito production over six years following the establishment of the *Anopheles darlingi* (A) and *Anopheles deaneorum* colony (B). Gray-shaded areas indicate yearly divisions, while gray-filled areas highlight months with mosquito production.

This protocol has been validated through the sustained production of adult mosquitoes over multiple years since colony establishment. Over the past six years, the mean monthly production was 22,753 *An. darlingi* adult mosquitoes and 7,906 *An. deaneorum* adult mosquitoes, as illustrated in Figure 8. The mean pupal mortality rate was 2.9% for *An. darlingi* and 5.4% for *An. deaneorum* (Table S10).

Additionally, several studies have utilized these colonies, demonstrating their reliability and research applicability [5,15,37]. The DMFA protocol has been thoroughly validated and applied in research investigating *P. vivax* infections [24,38].

## Supplementary information

The following supporting information can be downloaded here:

1. Table S1. Time schedule for *Anopheles darlingi* and *Anopheles deaneorum* mass-rearing based on insectary data (PIVEM).

2. Table S2. Water temperature register for rearing pans based on insectary data (PIVEM).

3. Table S3. Pupae register control based on insectary data (PIVEM).

4. Table S4. Badge for cage ID: pupae, blood feeding, and oviposition registration based on insectary data (PIVEM).

5. Table S5. Register of blood feeding and oviposition routine based on insectary data (PIVEM).

6. Table S6. Oocyst counts from seven independent infections (DMFA) between generations of *Anopheles darlingi.*

7. Table S7. Sporozoite counts from seven independent infections (DMFA) across generations of *Anopheles darlingi.*

8. Table S8. Data from each independent DMFA of *Anopheles darlingi.*

9. Table S9. General results of *Plasmodium vivax* susceptibility between *Anopheles darlingi* generations.

10. Table S10. Monthly production data of *Anopheles darlingi* and *Anopheles deaneorum* mosquitoes: General overview
